# Beyond the Bottleneck: Predicting Regeneration Potential in Sunflower Through Integrated Morphological and Statistical Profiling

**DOI:** 10.3390/ijms27020809

**Published:** 2026-01-14

**Authors:** Kimon Ionas, Mirjana Vukosavljev, Emilija Bulić, Aleksandra Radanović, Siniša Jocić, Ankica Kondić-Špika, Dragana Miladinović

**Affiliations:** Institute of Field and Vegetable Crops, National Institute of Republic of Serbia, Maksima Gorkog 30, 21000 Novi Sad, Serbia; kimon.ionas@ifvcns.ns.ac.rs (K.I.); mirjana.vukosavljev@ifvcns.ns.ac.rs (M.V.); emilija.bulic@ifvcns.ns.ac.rs (E.B.); aleksandra.dimitrijevic@ifvcns.ns.ac.rs (A.R.); sinisa.jocic@ifvcns.ns.ac.rs (S.J.); ankica.spika@ifvcns.ns.ac.rs (A.K.-Š.)

**Keywords:** sunflower regeneration, regeneration protocol, predictive morphological markers, genotype-by-medium interaction, temporal morphogenesis, sucrose concentration, multivariate analysis (hierarchical clustering)

## Abstract

This study presents the first integrated analysis of genotype–medium interactions and temporal morphogenesis profiling in sunflower regeneration. It aims to characterize genotype-specific responses, identify predictive morphological markers, and develop a scalable framework for breeding and transformation. Eighteen sunflower genotypes were evaluated to assess organogenic performance. The model genotype Ha-26-PR was used for a complementary experiment, testing varying sucrose concentrations to examine their influence on morphogenic outcomes. Hierarchical Cluster Analysis (HCA), guided by the Elbow method, identified four optimal clusters (K = 4). These aligned with three biologically meaningful categories: High Regenerators (Cluster 1), Moderate/Specific Regenerators (Clusters 2 and 3), and Non-Regenerators (Cluster 4). On S1 medium, NO-SU-12 and AS-1-PR showed superior shoot regeneration, while on R4 medium, HA-26-PR-SU and NO-SU-12 performed best. Genotypes such as NO-SU-12 and AS-1-PR consistently excelled across both media, whereas AB-OR-8 and FE-7 remained non-regenerators. Medium R4 supported superior regeneration, primarily through root formation, while S1 failed to induce roots in any genotype, highlighting the importance of hormonal composition. Although sucrose promoted callus induction, it did not trigger organogenesis. Callus was consistently present across media and time points, but its correlations with shoot and root formation were weak and temporally unstable, limiting its predictive value. Root formation at 14 days (Root 14D) emerged as a robust early predictor of organogenic success. This integration of morphological, temporal, and statistical analyses offers a genotype-tailored regeneration framework with direct applications in molecular breeding and CRISPR/Cas-based genome editing.

## 1. Introduction

Sunflower (*Helianthus annuus* L.) is one of the main oilseed crops cultivated worldwide at 30.14 million hectares and producing 58.57 million tons of seeds [1]. The total world sunflower seed production is used almost exclusively for oil extraction, providing 9% of the total global volume [2]. Sunflowers grow across diverse agroecological zones and tolerate drought moderately, making them promising future oil crops under global environmental change [3]. Yet, despite their market importance and adaptability, sunflowers remain one of the only major oil crops without authorized genetically modified (GM) varieties [4]. Recently, the clustered regularly interspaced short palindromic (CRISPR)/CRISPR-associated (Cas) systems have emerged as a promising approach for genome engineering in a variety of plant species. Although CRISPR-based technologies for targeted genome editing and gene regulation are advancing, their efficiency remains relatively low in plants. This limitation is especially critical for sunflowers, whose recalcitrance to in vitro regeneration and genetic transformation results in poor tissue culture response, making transgenic development and CRISPR/Cas9 implementation especially challenging [5,6,7]. Recent reviews have summarized the current status of haploid induction in sunflowers. They highlight γ-irradiated pollen as the most efficient method, while conventional in vitro approaches remain limited by low embryogenic response. Alternative strategies, including distant hybridization and CENH3-based manipulations, are emerging as promising directions [8]. These limitations underscore the need for improved regeneration systems, which our study directly addresses. Alternative approaches, such as immature embryo rescue, have recently been applied for inbred line development in sunflowers [9], highlighting complementary strategies to overcome regeneration bottlenecks. Therefore, a well-established plant regeneration protocol is often a prerequisite for genetic transformation. In sunflowers, regeneration strongly depends on genotype, media composition, and the specific combination and concentration of plant hormones [3,7,10]. Because of these intricacies, it is imperative to develop a regeneration protocol with high success rates for a variety of different genotypes and the right combination of media components. Recent advances in sunflower tissue culture have explored organogenesis and somatic embryogenesis in wild relatives [11]. Agrobacterium-mediated transformation has been successfully applied in elite cultivars [10], while regeneration from leaf explants has been used in endangered species [12]. Despite these developments, sunflowers remain recalcitrant due to genotype dependence, poor embryogenic response, and limited hormonal sensitivity. Many explants produce callus without organogenesis [5], underscoring the need for improved protocols that integrate physiological and cellular insights into regeneration pathways.

In vitro regeneration remains a foundation of modern plant biotechnology, providing a controlled platform for propagation, genetic transformation, and conservation. Among the medium components, sucrose plays a particularly complex role. Beyond serving as a carbon source, sucrose functions as an osmotic regulator and signaling molecule that directly influences morphogenetic decisions [13]. Moderate sucrose concentrations promote shoot and root induction, while excessive levels often lead to callus proliferation without organogenesis or vitrification [14,15]. Conversely, insufficient sucrose impairs root formation and delays differentiation [16]. These effects are strongly genotype-dependent, underscoring the need for tailored protocols that balance hormonal ratios with optimal sugar supply [17,18].

The successful in vitro regeneration of sunflowers remains a critical bottleneck, with consistent and high-frequency organogenesis as the central challenge. This difficulty stems from two tightly linked factors: the pronounced variability among genotypes and the delicate responsiveness of explants to their culture environment. Even under standardized conditions, small shifts in hormonal balance or medium composition can lead to divergent outcomes, making regeneration both unpredictable and genotype-dependent [19,20]. The core of this challenge lies in the Genotype × Environment (G × E) interaction. While superior genotypes possess the innate genetic competence for regeneration, this potential must be precisely unlocked by the culture medium [21]. A primary chemical agent in this environment is sucrose, which functions not only as the essential carbon source but also as a powerful osmotic regulator and a signaling molecule that directly impacts morphogenetic decisions [22,23]. However, the optimal concentration of sucrose—and indeed, the entire hormonal balance—is often genotype-dependent, leading to inconsistent results when a universal protocol is applied [24]. Furthermore, most studies rely on a simple assessment of the final regeneration percentages, overlooking the temporal aspect of morphogenesis. Tracking the sequence and efficiency of formation emergence (callus formation at 7 days, shoot and root induction at 14 days, and final outcome at 21 days) is critical for identifying early predictive markers. Relying solely on callus formation as an indicator has proven unreliable, as many lines successfully produce callus but fail to progress to organogenesis, suggesting a block in downstream differentiation pathways [25]. 

This study addresses the critical bottleneck of in vitro regeneration in sunflower by establishing a data-driven, genotype-specific framework. The overall goal was to identify and classify the inherent regeneration potential across diverse Helianthus annuus genotypes to facilitate their use in breeding and transformation pipelines. Specifically, we aimed (1) to evaluate the temporal morphogenic dynamics of 18 genotypes under a two-stage regeneration protocol (S1 → R4) and varying sucrose concentrations, and (2) to apply multivariate statistical methods (HCA and PCA) to group genotypes into biologically meaningful clusters. Based on the limited predictive value of callus formation, we hypothesize that mid-stage organogenic traits, such as shoot and root emergence at 14 days, provide more reliable markers of regeneration success than early callus induction.

## 2. Results

### 2.1. Regeneration Response: Genotype-by-Medium Interaction and Temporal Profiling

#### 2.1.1. Temporal Profiling and Hierarchical Clustering of Regeneration Formations

**Figure 1:**
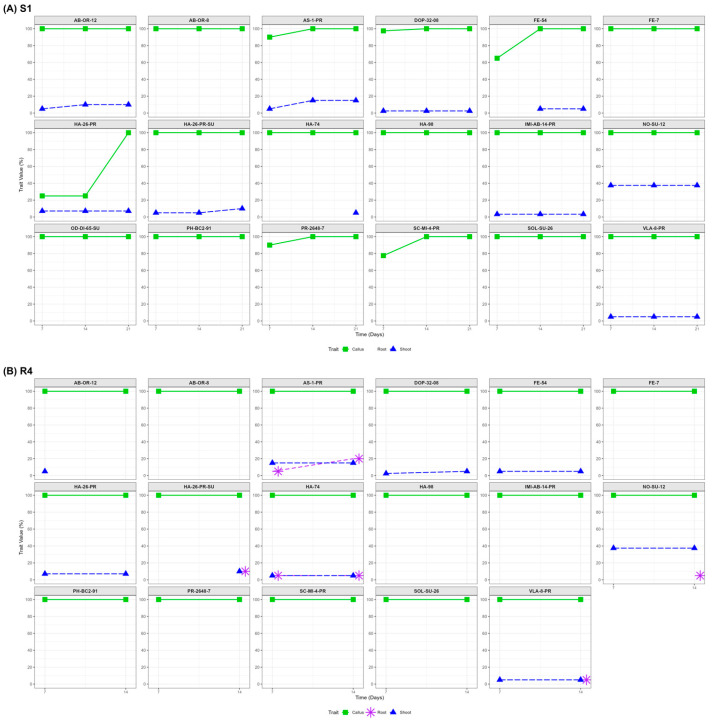
Temporal Regeneration Dynamics across 18 Sunflower Lines cultured on S1 medium for 21 days (**A**), and R4 medium for 14 days (**B**). Panel A represents lines cultured on S1 medium, where no root formation was observed, and resistance profiles are limited to shoot and callus formation. Panel B shows lines grown on R4 medium, which supported coordinated development of the shoot and roots over time. Colored lines indicate formation-specific resistance rates, revealing genotype-dependent differences in organogenic timing and intensity across substrates.

Regeneration-related formations were monitored over time (Figure 1) across genotypes cultured on S1 (7D, 14D, 21D) and R4 (7D, 14D) media, revealing distinct temporal trajectories and medium-specific responsiveness. On S1 (Figure 1A), callus formation peaked early (7D) and remained high. Shoot and root formations emerged later, with genotypes like AS-1-PR and AB-OR-12 showing increased organogenesis by 21D. In contrast, genotype NO-SU-12 exhibited shoot formation starting at 7D, which was sustained through the 21D measurement. 

On R4 (Figure 1B), callus induction was similarly rapid, but shoots and roots appeared earlier and more consistently. Lines like NO-SU-12, HA-26-PR-SU, and AS-1-PR demonstrated coordinated shoot and root formation by 14D.

Callus formation showed weak correlations with shoot and root formation across both media. Mid-to-late induction stages (14D for R4 and 21D for S1) corresponded to the highest observed organogenic percentages.

**Figure 2:**
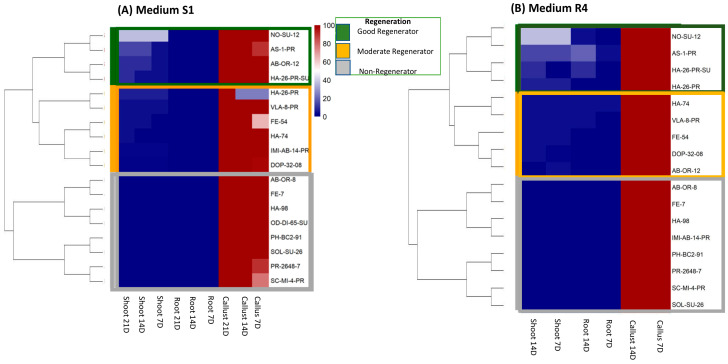
Hierarchical clustering of regeneration profiles in sunflower lines on media (**A**) S1 and (**B**) R4. The heatmap displays genotype-specific variation in callus, root, and shoot formation at 7, 14, and 21 days (D) post-induction on S1 and 7 and 14 days (D) post-induction on R4. Formation intensity is represented by a blue-to-red gradient, allowing visual comparison of temporal dynamics and phenotypic profiles across treatments. Distinct clusters corresponding to high-, moderate-, and non-regenerators are outlined in green, orange, and gray and labeled accordingly. A dendrogram is shown to the left of each heatmap to illustrate clustering of genotypes according to regeneration potential.

Hierarchical clustering revealed distinct genotype groupings on S1 and R4 media, with notable shifts in cluster membership between conditions (Figure 2). Although both media produced three major clusters, their composition varied, reflecting genotype-specific responsiveness to medium composition.

On S1 medium (Figure 2A), Cluster I comprised non-regenerating genotypes such as AB-OR-8 and FE-7, characterized by strong callus formation and the complete absence of shoot and root. Cluster II included moderate responders like HA-74 and HA-26-PR, which exhibited gradual formation expression over time. Cluster III grouped high regenerators such as NO-SU-12 and AS-1-PR, marked by early and sustained shoot development.

**Figure A1:**
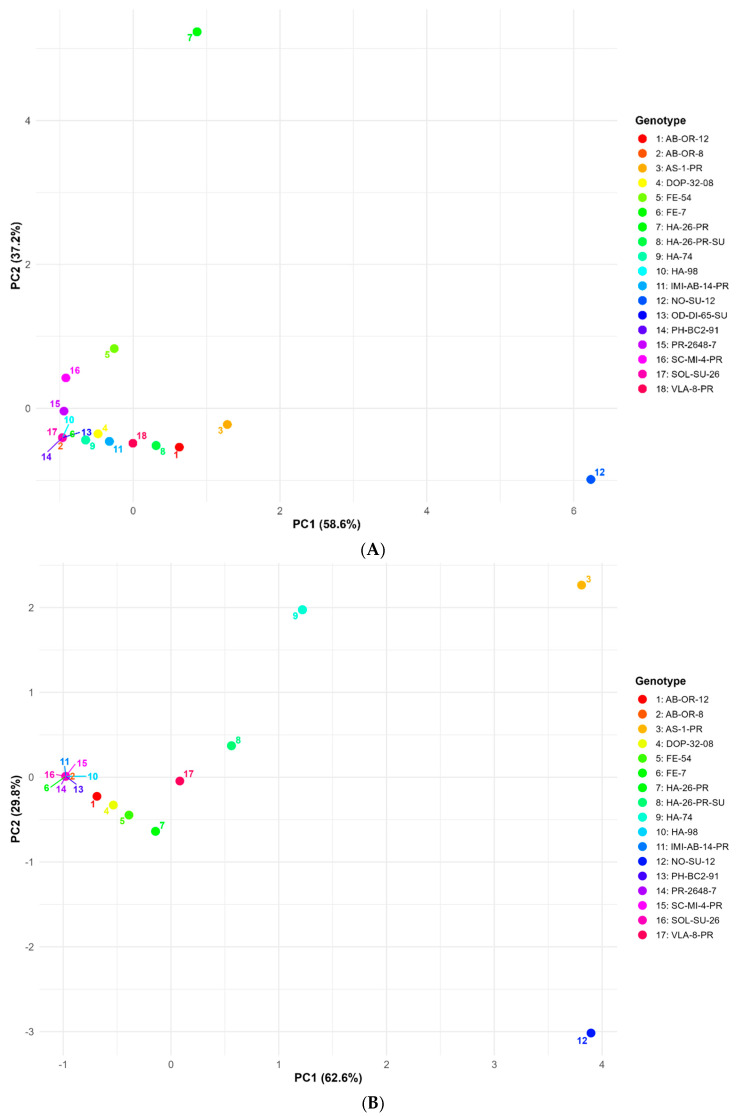
Distribution of 18 sunflower genotypes based on regeneration outcomes in S1 (21 days) and R4 (14 days) media. (**A**) Medium S1 shows the performance of genotypes in the S1 generation, where the first two principal components (PC1 and PC2) account for 58.6% and 37.2% of the total variance, respectively. (**B**) Medium R4 represents the genotypic performance in the R4 generation, with PC1 and PC2 explaining 62.6% and 29.8% of the variance, respectively. Each point represents a single genotype, and colors correspond to the genotype ID as detailed in the legend.

Clustering on R4 medium (Figure 2B) revealed a reorganization of genotypes. AB-OR-8 remained in the non-regenerating group, while AB-OR-12 shifted to a moderate cluster due to reduced shoot formation. Genotypes NO-SU-12, As-1-PR, and HA-26-PR-SU retained high regenerative profiles across both media. To further validate and visualize these genotypic relationships and shifts in performance, a complementary Principal Component Analysis (PCA) was conducted (Appendix A, Figure A1). This analysis strongly supported the clustering results by revealing genotype-level separation along principal components, with NO-SU-12 consistently positioned in regions associated with high regenerative capacity. The high cumulative variance explained by the first two components suggests clear separation and clustering of genotypes based on their regeneration ability.

**Figure 3:**
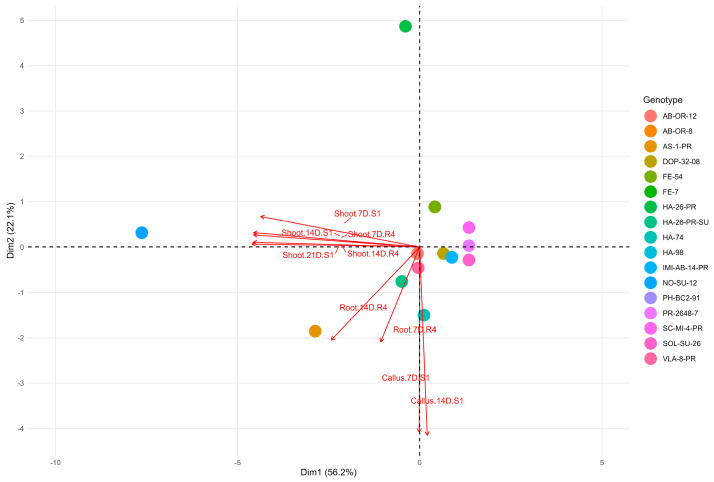
Principal Component Biplot for Combined Regeneration Profiles. The plot shows the projection of all 18 genotypes and all measured morphological traits (S1 and R4 medium formations) onto the first two principal components (PC1, 56.2%; PC2, 22.1%). Vectors represent trait correlations, and points represent genotype positions. The labels are defined by the formation type, time point, and medium used: S1 indicates cultivation on the Initial Shoot Induction Medium (Medium S1), R4 indicates cultivation on the Rooting Medium (Medium R4). Callus, Root, Shoot: The type of organogenic outcomes; 7D, 14D, 21D: Days of cultivation on the specific medium.

To further identify the fundamental morphological markers driving the regeneration process across both culture phases, a Principal Component Analysis (PCA Biplot) was performed on the entire combined multi-formation dataset (Figure 3). 

The analysis revealed a clear genotype-level separation based on morphogenic traits across media and time points. The first two principal components (Dim1 = 56.2%, Dim2 = 22.1%) jointly explained 78.3% of the total variance, indicating strong dimensional reduction and trait-driven clustering. PC1 (67.0% variance) represented the dominant dimension, exhibiting a strong correlation with the pace and intensity of shoot formation. This established PC1 as the primary statistical marker of overall regenerative competence. Crucially, PC2 (12.5% variance) emerged as a significant secondary dimension, primarily associated with root formation. PC1 was highly correlated with shoot formation, while PC2 was primarily associated with root formation, indicating their statistical separation in the dataset. The PCA results, showing distinct contributions of PC1 (Shoot) and PC2 (Root), support the utility of a two-stage S1 → R4 protocol.

Conversely, callus formations showed a weaker vector magnitude and orthogonal orientation, indicating limited contribution to genotype separation and reinforcing its low predictive value. The spatial distribution of genotypes relative to trait vectors supports the earlier clustering results and highlights the biological relevance of mid-stage organogenic traits in defining regeneration potential.

#### 2.1.2. Correlation of Regeneration Formations on S1 and R4 Media

**Figure 4:**
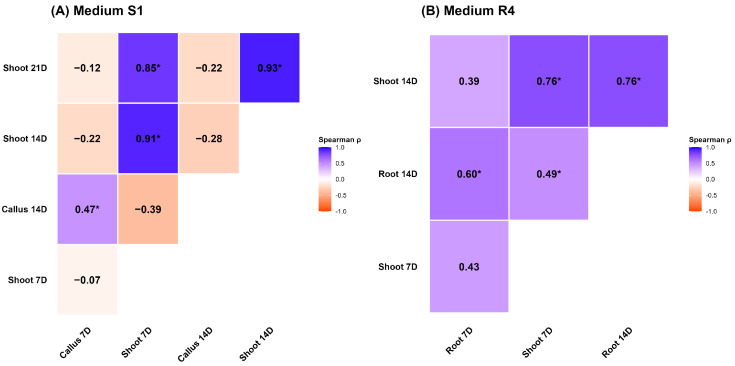
Correlation analysis of regenerative structures in sunflower genotypes. Panel (**A**) shows Spearman correlation heatmaps for genotypes grown on S1 medium for 21 days, with data collected at 7, 14, and 21 days post-induction. Panel (**B**) displays correlations for genotypes cultured on R4 medium for 14 days, with data collected at 7 and 14 days post-initiation. Each triangular heatmap represents pairwise correlations between callus, shoot, and root formations over time. Correlation coefficients (ρ) range from −1 (strong negative correlation, dark blue) to +1 (strong positive correlation, dark red). Formation columns with zero variance were automatically excluded from the analysis. Statistical significance is indicated by asterisks: * *p* < 0.05.

Correlation analysis revealed medium-specific patterns in formation interdependence across sunflower genotypes. On S1 medium (Figure 4A), the hormonal balance establishes a highly selective environment for shoot development. Root formations were excluded from the S1 correlation matrix because root formation was absent on this medium for all tested genotypes. The matrix reflects that the substrate selectively supported shoot formation. Despite the inconsistent correlation of callus, the predictive value of shoot formations themselves was remarkably high. The analysis showed strong, significant positive correlations among all measured shoot time points. Early shoot emergence at 7 days was highly and significantly correlated with the final shoot percentage at 21 days on S1 medium. In terms of early-stage associations, the link between Callus 7D and Shoot 7D (*ρ* = −0.07) suggested a partial competitive or sequential relationship in developmental pathways, possibly reflecting initial hormonal responsiveness or tissue competence. This relationship diminished or reversed as the culture progressed. At 14 days, the correlation between callus and shoot regeneration percentage was *ρ* = −0.28, indicating that callus formation alone is insufficient to predict shoot induction outcomes.

In stark contrast, on R4 medium (Figure 4B), the coordination of regeneration outcomes was significantly more robust and predictive, particularly at day 14 post-initialization (14D). Root 14D showed exceptionally strong and highly significant associations with Shoot 14D (*ρ* = 0.76 *, *p* < 0.05), clearly highlighting this time point as a critical window for synchronized organogenic activation. Furthermore, Shoot 7D was highly correlated with Shoot 14D (*ρ* = 0.76 *, *p* < 0.05). This establishes Shoot 7D as a key early morphological marker for predicting final regeneration success on the R4 medium. Callus regeneration, conversely, exhibited weak correlations with both root and shoot formation (e.g., Callus 14D vs. Shoot 14D, *ρ* = 0.17).

#### 2.1.3. Stratification of Genotypes Based on Regeneration Performance

**Figure A2:**
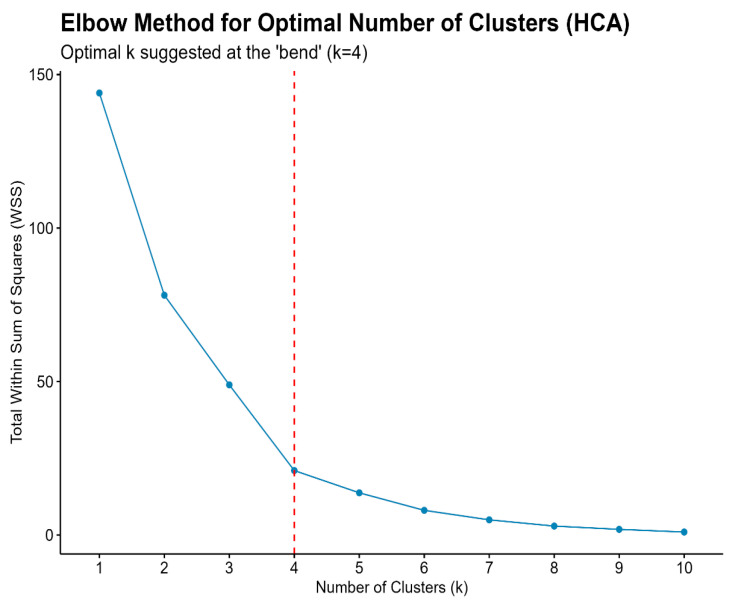
Optimal Number of Clusters Determined by Elbow Method. Elbow plot illustrating the total within-cluster sum of squares (WSS) across increasing numbers of clusters (k = 1–10) for hierarchical clustering analysis. The sharp inflection point at k = 4 indicates the optimal number of clusters, beyond which additional clusters yield diminishing returns in explanatory power.

To capture genotype-specific differences in regenerative capacity, Hierarchical Cluster Analysis (HCA) and Z-score heatmapping were applied to multi-formation regeneration profiles. Both the Elbow method (WSS plot) and the Silhouette analysis were employed to validate the optimal number of clusters (K) for the HCA. The WSS plot indicated a clear point of diminishing returns (the ‘elbow’) at K = 4, beyond which the reduction in variance was marginal (Appendix A, Figure A2). This choice was corroborated by the Silhouette analysis, which yielded an average width of 0.54, suggesting a reasonable separation and cohesiveness among the four resulting genotype groups. These complementary analyses confirmed the robustness of using four distinct clusters for the final stratification of the regeneration profiles.

**Figure 5:**
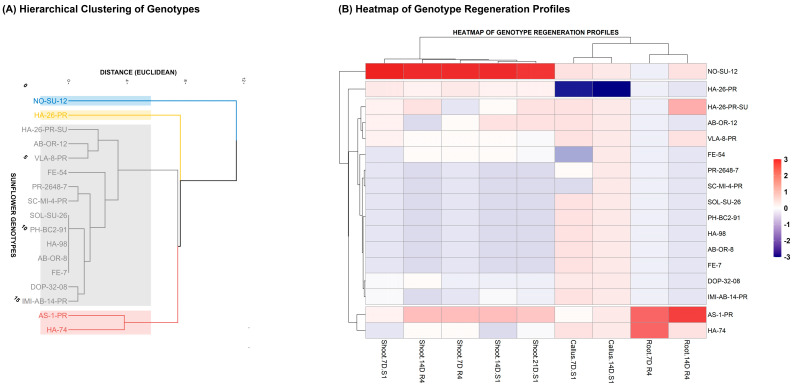
Stratification of Genotypes Based on Regeneration Performance. (**A**) Dendrogram of Genotype Regeneration Profiles (HCA) illustrates the grouping of 18 sunflower genotypes into three main clusters: Cluster 1—blue (Good Regenerators), Cluster 2—yellow, Cluster 3—red (Moderate Regenerators), and Cluster 4—gray (non-regenerators) based on the Euclidean distance of their multi-formation performance. (**B**) Heatmap of Genotype Regeneration Profiles visualizes the standardized (Z-score) performance for three aggregated categories (Callus, Root, and Shoot). Intense red coloration signifies performance significantly above the population mean (Z > 0), while intense blue indicates performance below the mean (Z < 0). The heatmap labels are defined by the formation type, time point, and medium used: S1 indicates cultivation on the Initial Shoot Induction Medium (Medium S1), R4 indicates cultivation on the Rooting Medium (Medium R4); Callus, Root, Shoot: The type of organogenic outcomes; 7D, 14D, 21D: Days of cultivation.

Hierarchical clustering (Figure 5A) partitioned the population into four statistically distinct clusters. For biological interpretation, these were consolidated into three meaningful categories: Cluster 1 (blue) comprised High Regenerators such as NO-SU-12 and AS-1-PR; Clusters 2 (yellow) and 3 (red) grouped Moderate/Specific Responders including HA-26-PR and HA-74; and Cluster 4 (gray) encompassed Non-Regenerators such as AB-OR-8 and PH-BC2-91. Notably, NO-SU-12 formed an isolated branch, indicating exceptional morphogenic competence and statistical separation from the rest of the population. 

The accompanying Z-score heatmap (Figure 5B) visualizes standardized performance across shoot, root, and callus formations at key time points. High-performing genotypes exhibited strongly positive Z-scores (red) in shoot and root categories, while Non-Regenerators showed negative scores (blue), particularly in shoot formation. Although callus was consistently present, its low predictive value was reflected in weak or neutral Z-scores across genotypes.

Together, these visualizations confirm the robustness of genotype stratification and highlight shoot formation as the dominant trait driving regenerative success.

#### 2.1.4. Temporal Dynamics of Regeneration

Regeneration-related formations were monitored over time (Figure 5) across genotypes cultured on S1 (7D, 14D, 21D) and R4 (7D, 14D) media, revealing distinct temporal trajectories and medium-specific responsiveness. On S1 (Figure 5A), callus formation peaked early (7D) and remained high. Shoot and root formations emerged later, with genotypes like AS-1-PR and AB-OR-12 showing increased organogenesis by 21D. In contrast, genotype NO-SU-12 demonstrated early and sustained shoot formation throughout the culture period, indicating a robust and stable morphogenic potential under S1 conditions.

Across both media, early callus formation was not a reliable predictor of organogenesis. Instead, mid-to-late induction stages—particularly 14D for R4 and 21D for S1—proved critical for evaluating regenerative potential. Lines with synchronized shoot and root emergence across time points and media, such as NO-SU-12, HA-26-PR-SU, and AS-1-PR, exhibited consistent morphogenic profiles.

#### 2.1.5. Non-Parametric Analysis of Shoot Regeneration Across Genotypes and Media

Regeneration responses varied across sunflower genotypes and media, with most genotypes exhibiting consistent morphogenic activity by day 14 after culture initialization. Non-parametric analysis using the Kruskal–Wallis rank sum test revealed no statistically significant differences in regeneration either across genotypes (*p* = 0.4544) or between media types S1 and R4 (*p* = 0.8973). This suggests that regeneration was broadly comparable under the tested conditions. Despite the lack of statistical significance, individual genotype performance showed clear biological variation.

**Figure 6:**
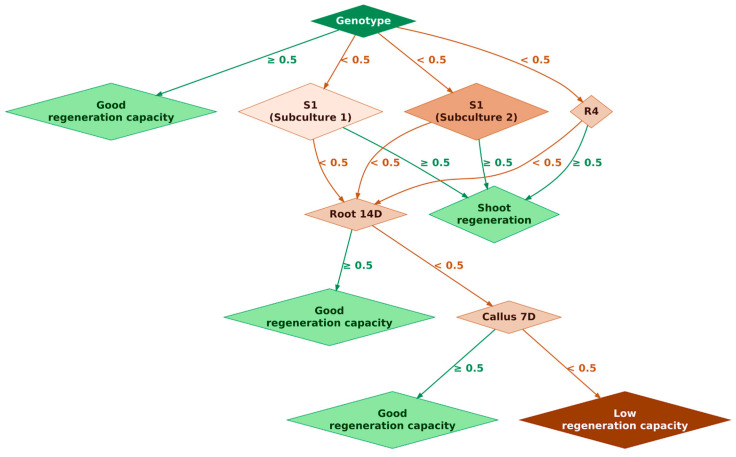
Decision tree-based pipeline illustrating genotype-based prediction of regenerative outcomes using key experimental parameters. The model was constructed using genotype identity, medium composition (subculture type), regeneration timing, and trait-specific emergence as predictive variables. The tree was trained using the Gini impurity criterion with a complexity parameter (cp) of 0.01, achieving an in-sample classification accuracy of 94.7%. Each node represents a decision point based on a threshold value (< or ≥0.5), guiding the classification toward either stable or unstable regenerative outcomes. The root node (“Genotype”) serves as the primary classifier, separating genotypes with high regenerative potential (≥0.5) from those requiring further evaluation (<0.5). Genotypes with strong baseline performance are classified early as having “Good regeneration capacity.” For genotypes with lower initial potential, the next decision nodes—“S1 Subculture 1”, “S1 Subculture 2”, and “R4”—represent different culture conditions that influence the regenerative trajectory. Node color intensity for subculture conditions (S1 Subculture 1, S1 Subculture 2, and R4) is scaled according to regeneration success rates. Lighter shades indicate higher percentages of regenerated plants, and darker shades represent lower regeneration outcomes. Favorable subculture responses lead directly to shoot formation (“Shoot regeneration”), while suboptimal responses prompt further evaluation. From the shoot regeneration node, the model assesses root formation at day 14 after culture initiation (“Root 14 D”), a critical temporal marker for regenerative stability. If root formation is absent or delayed, the model proceeds to evaluate callus development at day 7 (“Callus 7 D”), an early indicator of morphogenic potential. The absence of both traits leads to classification as “Low regeneration capacity”. Conversely, early emergence of root and callus traits supports a classification of “Good regeneration capacity”.

To further explore the determinants of regenerative success, a decision tree classification model was constructed using key experimental parameters: genotype identity, nutrient composition (different media), regeneration timing, and the presence of shoots and roots (Figure 6). The model achieved an in-sample classification accuracy of 94.7%, with high precision and recall confirmed by confusion matrix analysis, indicating strong separation between stable and unstable regenerants. 

The decision tree structure revealed genotype identity as the most influential initial classifier. Genotypes exhibiting high regenerative competence were promptly assigned to the “Good regeneration capacity” category. For lines with lower baseline potential, medium composition became the critical branching factor. Suboptimal nutrient concentrations led to further splits based on Root development at day 14 after of culturing, which in turn predicted early callus formation or low regenerative outcomes. Notably, genotypes FE-54, HA-26-PR, and HA-26-PR-SU displayed distinct behavior under hypocotyl-based protocols. The presence of nutrients in the medium, combined with shorter regeneration time, was associated with phenotypic stability, while delayed shoot formation and absence of roots predicted instability.

### 2.2. Regeneration Response Under Sucrose Variation

The influence of sucrose concentration on morphogenic responses was assessed through two complementary experiments. Across both experiments, callus formation was the dominant and consistent morphogenic response, while root and shoot regeneration were absent under all tested conditions. 

#### 2.2.1. Preconditioning on MS Medium

**Figure 7:**
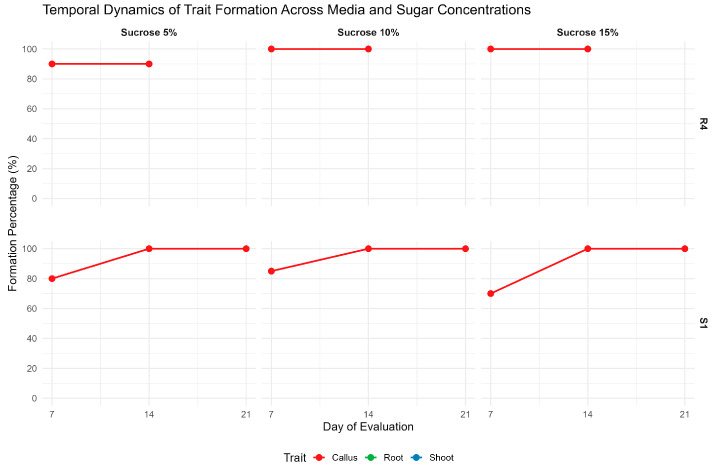
Temporal Dynamics of Regeneration Under Varying Sucrose Concentrations (5%, 10%, 15%) on MS medium. Regeneration outcomes formation was monitored at 7-, 14-, and 21 days post-induction on S1 medium and 7- and 14 days post-induction to R4, with panels representing callusregeneration in each medium. Shoot and root regeneration were not observed under these conditions.

Hypocotyl explants originated from seeds that were germinated on MS medium with 5%, 10%, or 15% sucrose, exhibiting high callus regeneration upon transfer to S1 medium and then on R4 (Figure 7). 

On S1 medium, callus induction varied slightly across sucrose concentrations. At 5% sucrose, 80% of explants formed callus by day 7, increasing to 100% by day 14. At 10% sucrose, induction was 85% on day 7 and reached 100% by day 14. At 15% sucrose, initial callus formation was lower (70% on day 7), but also reached 100% by day 14. 

On R4 medium, callus formation at 5% sucrose was reduced to 90%, as some explants that previously formed callus on S1 (21 days) no longer retained it at day 7 on R4. In contrast, callus induction at 10% and 15% sucrose remained stable at 100%, with no variation between day 7 and day 14. No shoot or root formation was observed on R4 at any sucrose concentration or time point. This indicates that sucrose alone does not promote organogenesis under these conditions.

#### 2.2.2. Direct Sucrose Variation on S1 Medium

**Figure 8:**
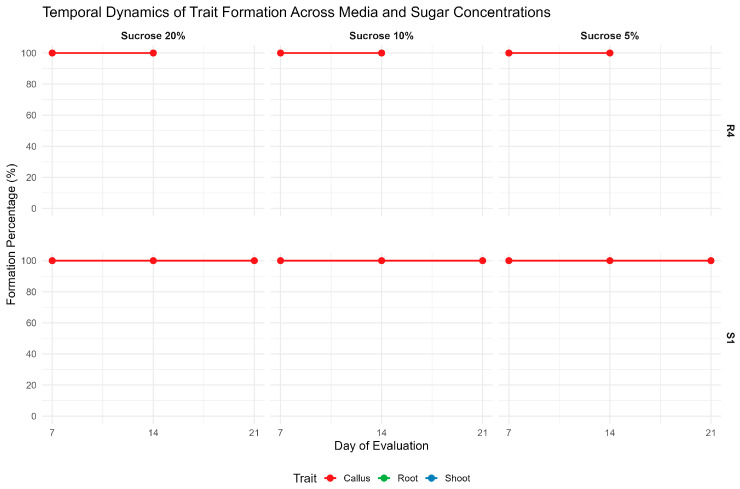
Temporal Dynamics of Regeneration Under Varying Sucrose Concentrations (5%, 10%, 20%) on S1 Media. Regeneration outcome formation was monitored at 7-, 14-, and 21 days post-induction on S1 medium and 7- and 14 days post-induction on R4 medium, with panels representing callus responses across treatments. Shoot and root responses were not observed under these conditions. The figure highlights the predominance of callogenesis in the absence of functional organogenic differentiation under the tested sucrose regimes.

In the second experiment, hypocotyl explants were cultured on S1 medium with 5%, 10%, or 20% sucrose showed similar trends. Figure 8 illustrates that, despite sustained and near-complete callus formation across treatments, these conditions failed to induce stable shoot or root organogenesis, visually reinforcing the quantitative regeneration data. Callus formation remained close to 100% across all concentrations and time points (days 7–21). However, root and shoot regeneration were minimal or entirely absent, regardless of sucrose level. The data suggest that these concentrations do not promote organogenesis but primarily support the maintenance of callogenic tissue.

## 3. Discussion

To our knowledge, this is the first study integrating genotype-by-medium interaction analysis with temporal morphogenesis profiling to identify early predictive markers of regeneration capacity in sunflowers. By linking morphological dynamics with multivariate analyses, this study provides a data-driven framework for improving genotype selection and regeneration protocol optimization. This section first summarizes the main regeneration patterns across genotypes and culture media, then interprets the biological basis of genotype-by-medium interactions, and finally considers the practical implications of early predictive morphogenic traits for sunflower regeneration.

The results demonstrate pronounced genotype-dependent differences in regeneration responses across the two media, S1 and R4. In general, S1 promoted callus formation and occasional shoot induction due to the auxin–cytokinin balance, whereas R4 was designed to favor root formation under cytokinin-dominant conditions [26]. Across both media, most genotypes formed callus early and robustly, reaching peak values by day 7, consistent with auxin-induced cellular reprogramming. However, the lack of subsequent shoot formation in certain lines shows that callus induction alone is not predictive of organogenic success, echoing previous observations [27]. Our results confirm that regeneration outcomes depend on cellular competence and hormonal sensitivity, consistent with established principles of plant totipotency [28,29]. The contrasting responses on S1 and R4 reflect differences in hormonal composition: S1 favored shoot induction, while R4 enabled coordinated shoot and root development [26,27]. At the cellular level, regeneration requires dedifferentiation, cell cycle reactivation, and transcriptional reprogramming, with callus representing an intermediate pluripotent state. Key regulators such as WUSCHEL and BABY BOOM, together with chromatin remodeling and stress-responsive signaling, promote meristem identity and totipotency [28,29]. Genotype-dependent variation arises because not all cells express totipotency equally. Parenchyma cells readily form callus but rarely progress to organogenesis [19], whereas meristematic populations possess higher competence [30]. External cues such as wounding, stress, and hormone treatments further modulate competence by altering auxin–cytokinin gradients and metabolite redistribution [31,32]. In sunflowers, these principles are complicated by strong genotype dependence, with regeneration efficiency varying across explant types and hormonal regimes [33,34,35]. This variability underscores the need for reproducible, genotype-specific regeneration systems to advance breeding and transformation pipelines.

The Principal Component Analysis (PCA) provided a crucial, complementary validation of the clustering results, revealing the multi-formation basis for genotypic differentiation. Genotypes such as NO-SU-12, AS-1-PR, and HA-26-PR-SU expressed coordinated shoot and root development, proving to be strong regenerators. Their outlier profiles in PCA plots and strong inter-formation correlations reflect inherent organogenic competence, likely reflecting favorable hormonal sensitivity and transcriptional activation [36,37]. Specifically, the extreme and isolated position of genotype NO-SU-12 (point 12) on both S1 and R4 biplots is highly significant. On Medium S1, NO-SU-12 is positioned positively and distantly along PC1 (58.6% of variance), confirming that it is not merely a high regenerator, but a unique biological entity within the population. This separation is rooted in its distinct phenotype: the early and sustained dominance of shoot formation (as previously demonstrated in the temporal heatmaps). Since PC1 generally represents overall regenerative capacity, the extreme position of NO-SU-12 indicates that this genotype possesses a specific, highly efficient combination of formations contributing disproportionately to the total variance. This makes NO-SU-12 an ideal and statistically distinct candidate for further studies. Conversely, lines PH-BC2-91 and SC-MI-4-PR produced dense calluses but consistently failed to initiate shoots. This suggests a potential disruption of cytokinin-mediated induction pathways and highlights the necessity for genotype-specific hormonal calibration to overcome this developmental block [38,39]. Beyond conventional callus-based protocols, ovary transformation and related approaches have proven more effective in several *Asteraceae* species, offering significant advantages in terms of embryogenic response and stable regenerant production [40,41]. Although these methods are not yet widely applied in sunflowers, their success in related taxa highlights the potential for adapting such strategies to overcome current regeneration bottlenecks. Integrating insights from ornamental *Asteraceae* with sunflower regeneration studies may provide valuable directions for optimizing genotype-specific protocols and expanding the toolkit available for transformation and breeding applications.

Genotype IMI-AB-14-PR regenerated moderately on S1 but failed to respond on R4, suggesting a possible genotype-by-medium interaction that may be influenced by hormonal sensitivity [42,43]. Similarly, genotype HA-26-PR exhibited contrasting responses across media, highlighting its selective sensitivity to substrate composition. On R4, it initiated early shoot proliferation without root formation, suggesting partial activation of organogenic pathways. In contrast, S1 induced delayed yet sustained callus formation, with minimal shoot development. These divergent outcomes imply that HA-26-PR responds to medium-specific cues, possibly shaped by hormonal thresholds, underscoring the need to tailor regeneration protocols to genotype–medium interactions [44].

To further disentangle these genotype-specific responses and identify the traits driving regeneration success, multivariate statistical approaches were applied.

The rationale for utilizing a two-stage protocol (S1 → R4) is further supported by the findings of the Principal Component Analysis. The PCA demonstrated that the dominant shoot formation traits (S1 phase) drive most of the variance (PC1), while root formation traits (R4 phase) reside on the orthogonal secondary dimension (PC2).

This statistical orthogonality between the dominant shoot and root markers is critical. It indicates that the S1 and R4 media activate two different, yet complementary, morphogenetic programs. Had the R4 traits simply been a continuation of the S1 phase, their corresponding vectors would have been highly correlated with PC1. The observed independence, together with the failure of S1 medium to induce rooting in any genotype, confirms that the R4 medium is a necessary, specialized phase for successful regeneration, rather than merely a growth medium.

The statistical separation of the dominant shoot markers (PC1) and the root markers (PC2) suggests that rooting is an independent morphogenetic pathway that is not necessarily linked to a high shoot induction capacity. This result further supports the two-stage protocol, demonstrating that the R4 medium drives a distinct and specialized phase of organogenesis.

Beyond genotype classification, temporal analysis revealed that not all morphogenic traits contribute equally to successful regeneration.

Correlation analysis revealed that early callus formation (7D) did not predict shoot regeneration, particularly on S1 medium (*ρ* = −0.07). In contrast, on R4, root formation at 14 days correlated strongly with shoot development (*ρ* = 0.76), indicating a coordinated organogenic trajectory specific to this medium. These results position Root 14D as a reliable early predictor of regenerative success, consistent with findings in Arabidopsis and *Thottea siliquosa* [25,45].

Although later-stage callus formations have been linked to hormonal transitions and meristem-associated gene expression [25,45,46], our data revealed a strong negative correlation between callus formation at 14 days and shoot development. This suggests that, within our system, excessive or prolonged callus proliferation may inhibit organogenesis rather than support it. Such divergence from established models (e.g., *Populus alba*) may reflect species-specific dynamics, genotype-dependent hormonal thresholds, or medium-specific effects. These findings underscore the need to reassess the role of callus maturation in sunflower regeneration and caution against assuming universal developmental trajectories across systems.

R4 medium generally supported superior shoot regeneration across genotypes, likely due to a more favorable cytokinin-to-auxin ratio [47,48]. In contrast, S1 failed to induce root development, suggesting that its hormonal balance is suboptimal for complete organogenesis. Notably, explants transferred from S1 to R4 exhibited markedly improved shoot induction, indicating that R4 provides a more inductive environment during the critical reprogramming window.

The divergent correlation patterns between S1 and R4 confirm that regenerative coordination is both medium- and time-dependent. While S1 promotes callus proliferation without organogenic progression, R4 enables synchronized shoot and root development. The strong positive correlation between Shoot 14D and Root 14D on R4 (ρ = 0.76) highlights this time point as a key window for evaluating regenerative competence and selecting responsive genotypes. These medium-specific correlation patterns aligned with the genotype stratification observed in both hierarchical clustering and PCA. Genotypes exhibiting synchronized shoot–root development (e.g., NO-SU-12, AS-1-PR) consistently clustered together and occupied distinct PCA regions, confirming the biological relevance of formation coordination.

Hierarchical clustering separated genotypes into three categories—non-regenerators, moderate responders, and high regenerators—providing a practical framework for selection in breeding and transformation [49]. These genotype-specific profiles deepen understanding of morphogenetic plasticity in sunflowers and support the design of tailored regeneration systems applicable to other recalcitrant crops. Together, these observations indicate that regeneration outcomes in sunflower are primarily driven by genotype-specific sensitivity to hormonal environments rather than by callus induction per se.

Identifying early predictive formations such as Root 14D opens opportunities for marker-assisted selection, accelerating regeneration protocol development. The clear genotype-by-medium interactions observed highlight the importance of hormonal sensitivity and dynamic assessment rather than single-time-point evaluations. 

The Kruskal–Wallis test did not reveal statistically significant differences, likely reflecting the high biological variability and limited replication inherent to early-stage tissue culture experiments. Although univariate significance was not achieved, the clear biological divergence captured by multivariate analyses highlights the limitations of single-factor tests in such contexts [50]. Principal component and cluster analyses effectively resolved distinct genotype groupings, underscoring their robustness in detecting complex, multidimensional patterns. The multivariate clustering approach was supported by both internal and external validation metrics. Silhouette analysis (average width = 0.54) indicated moderate separation among genotype groups, while permutation-based bootstrap testing (AU > 95%) confirmed the statistical stability of key clusters. These complementary results reinforce the biological relevance and robustness of the genotype stratification derived from regeneration trait profiles. These discrepancies between statistical and biological resolution are common in tissue culture studies, where high variance and low replication often mask meaningful trends. The complementary use of univariate and multivariate approaches thus provides a more nuanced and comprehensive understanding of regeneration variability [28,51,52].

Both sucrose experiments—variation on MS medium during germination and on S1 medium—showed that sucrose concentration consistently supported callus formation but failed to induce organogenesis. Callus induction reached nearly 100% across treatments, indicating sufficient energy and osmotic support for dedifferentiation. Apart from minimal shoot initiation at 5% sucrose, no consistent root or shoot regeneration was observed. This aligns with Guleria and Kumar’s [53] findings, who noted that sucrose acts synergistically with hormones to drive organogenesis but alone is insufficient. Excessive sucrose can also disrupt cytokinin homeostasis and downregulate morphogenic genes, delaying organ formation [54]. 

Our results indicate that hormonal balance, rather than carbon source concentration, limits morphogenic progression. Both S1 and R4 may require further adjustment of cytokinin-to-auxin ratios to trigger organ differentiation. Environmental factors such as light quality, temperature, and explant orientation may also contribute to overcoming developmental constraints [55]. These findings highlight the need to integrate sucrose modulation with fine-tuned hormonal and environmental control to achieve complete regeneration in sunflower. 

Distinct genotype-dependent regeneration capacities were observed among sunflower lines. High-performing lines such as NO-SU-12 and AS-1-PR exhibited stable shoot and root induction, particularly on R4, confirming its favorable hormonal profile. Conversely, genotypes like FE-54 and HA-74 remained restricted primarily to callus formation, failing to progress significantly toward shoot or root organogenesis across the tested media. The observed genotype-specific limitations might suggest a genetically determined block in the downstream organogenesis pathway. Future studies would benefit from investigating the presence and expression levels of key morphogenic genes, such as WUSCHEL-related homeobox (WOX4), to molecularly define these observed differences in regenerative capacity [37].

Some genotypes (e.g., HA-26-PR-SU) maintained consistent regenerative responses across both media, indicating an adaptability valuable for breeding and transformation pipelines [5,47]. 

Overall, regeneration success in sunflowers results from the interplay of genotype, hormonal environment, and physiological state. Integrating these insights with transcriptomic and functional genomic tools may enable targeted improvements to regeneration systems [11]. Beyond conventional callus-based protocols, ovary transformation and related approaches have proven more effective in several *Asteraceae* species, offering significant advantages in terms of embryogenic response and stable regenerant production [40,41]. Although these methods are not yet widely applied in sunflowers, their success in related taxa highlights the potential for adapting such strategies to overcome current regeneration bottlenecks. Integrating insights from ornamental *Asteraceae* with sunflower regeneration studies may provide valuable directions for optimizing genotype-specific protocols and expanding the toolkit available for transformation and breeding applications.

Regeneration remains the primary bottleneck in sunflower transformation pipelines, particularly for CRISPR/Cas applications [56,57]. The genotype-specific regeneration profiles and early predictive markers identified here provide a practical decision framework for selecting transformation-competent lines, reducing experimental attrition.

Nevertheless, the modest number of genotypes and biological replicates may constrain statistical power. Expanding genotype diversity and incorporating molecular assays—transcriptomic, metabolomic, and epigenetic—could elucidate mechanisms underlying regeneration competence. Integrating morphological and molecular markers will strengthen selection strategies for large-scale propagation, stress adaptation, and advanced genome editing in sunflowers.

## 4. Materials and Methods

### 4.1. Regeneration of 18 Genotypes

**Figure 9:**
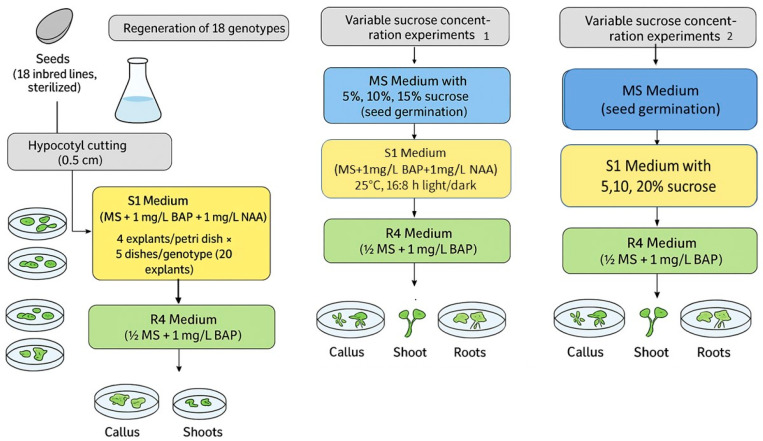
Schematic Flowchart of the Sunflower Regeneration Protocol and Variable Sucrose Trials.

Seeds from 18 different inbred lines from the Institute of Field and Vegetable Crops, Novi Sad (Serbia), were used in the study (Appendix A, Table A1). The entire experimental design, encompassing the two-stage regeneration protocol and variable sucrose trials, is schematically represented in Figure 9 for visual clarity. These seeds were sterilized according to the protocol proposed by Taški-Ajduković [58]. After sterilization, the seeds were placed in Erlenmeyer flasks with 100 mL of MS medium (Murashige and Skoog) [59]; 0.7% agar, pH 5.8. Four seeds were placed per Erlenmeyer flask, with three Erlenmeyer flasks set up for each genotype. Seeds were cultured for 7 days at 25 °C under a 16:8 h light/dark photoperiod. After 7 days, hypocotyl segments 0.5 cm long were cut and placed in a Petri dish with 15 mL of S1 medium (MS medium with 1 mg/L BAP and 1 mg/L NAA). Each Petri dish contained 4 explants, with 5 Petri dishes per genotype (20 explants in total). S1 medium is primarily used to induce callus formation of the explants, and in some cases, shoot formation, reflecting the balance of auxins and cytokinins. The explants were kept at 25 °C under a 16:8 h light/dark photoperiod for 21 days. The number of formed calluses, shoots, and roots of each explant was determined after 7, 14, and 21 days. After 21 days, calluses were transferred into new Petri dishes containing 15ml of R4 medium (modified MS medium with 1⁄2 macro elements and 1 mg/l BAP), 4 explants per Petri dish. R4 medium is designed mainly for the induction of root formation with the presence of only cytokinins. The explants were kept at 25 °C under a 16:8 h light/day photoperiod for 14 days. The number of calluses, shoots, and roots of each explant was determined after 7 and 14 days from placement on R4 medium.

**Figure 10:**
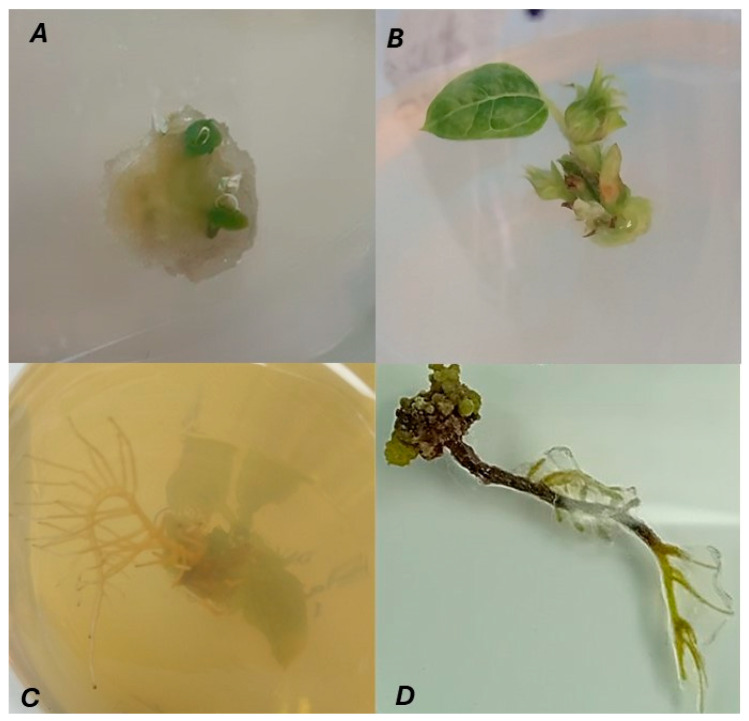
Representative stages of in vitro regeneration in sunflower. Sequential phases of morphogenic development observed during tissue culture of sunflower explants. (**A**) Initiation of shoot formation from callus tissue; (**B**) advanced shoot development with multiple leafy structures; (**C**) root induction; (**D**) regenerated plantlet with coordinated shoot and root structures.

Regeneration capacity was evaluated by direct visual inspection of each explant at defined time points (7, 14, and 21 days). For every explant, the presence or absence of morphogenic formations (callus, shoots, and roots) was recorded under standard culture conditions. Morphogenic formations were defined a priori: callus (new proliferative tissue), shoot (visible bud/leaf primordium), root (elongated white structure ≥ 3 mm). Since all experiments were conducted with biological replicates, regeneration capacity was expressed as the percentage of explants that successfully developed the respective formations within each treatment. Representative photographs were taken to document critical stages, and the key morphogenic phases—callus induction, shoot initiation, and root development—are illustrated in Figure 10.

### 4.2. Variable Sucrose Concentration in Regeneration Media

Genotype Ha-26-PR, our model genotype for tissue culture, was subsequently used in a separate set of trials. Both trials followed the regeneration protocol outlined previously.

Two complementary experiments were conducted to evaluate the influence of sucrose concentration on morphogenic responses in sunflower explants.

In the first experiment, seeds were germinated on MS medium with sucrose in three concentrations—5%, 10% and 15%. Then, similarly to the previous experiment, hypocotyl explants were cut and transferred on S1 medium for 21 days and then on R4 for 14 days. Monitoring occurred every 7 days (three time points during S1 culture and two time points during R4 culture).

In the second experiment, seeds were germinated on normal MS medium and then the hypocotyl explants were cultured on S1 medium containing 5%, 10%, or 20% sucrose for 21 days and then transferred on R4 medium for 14 more days. Regeneration outcomes were assessed over the same time intervals. This set up allowed for direct evaluation of sucrose impact on shoot-inductive conditions.

Across both experiments, regeneration responses were assessed for callus, root, and shoot formation. The combined approach enabled comparison between preconditioning effects and direct sucrose modulation within regeneration-specific media.

### 4.3. Statistical Analysis and Data Visualization

All statistical analyses and graphical visualizations were performed using RStudio (version 2025.5.1.513) [60] within the R programming environment. The analytical workflow combined descriptive and inferential approaches to assess the effects of genotype, medium composition, sucrose concentration, and time on regeneration formations.

Given that the data did not meet assumptions of normality, differences in regeneration capacity across genotypes and between media types were evaluated using the Kruskal–Wallis rank sum test, a non-parametric method selected for its robustness against deviations from normal distribution and unequal variances. 

To further explore regeneration formations interdependence across time points and media, Spearman’s rank correlation analysis was conducted. This non-parametric approach was selected due to the non-normal distribution of regeneration percentages and the presence of formations with zero variance (e.g., consistently 100% callus formation) to assess the strength and direction of monotonous relationships among the formations. Principal Component Analysis (PCA) was employed to reduce dimensionality and visualize genotype-specific variation in regeneration responses. Hierarchical clustering (complete linkage, Euclidean distance) was used to group genotypes based on regeneration formation expression profiles. The optimal number of clusters (K) was determined using two complementary methods: the Elbow method (WSS plot) and the Silhouette analysis (Figure A1, Appendix A Data). Both methods consistently pointed to an optimal partition of the population into four clusters (K = 4), which was subsequently adopted for the final clustering visualization. Clustering was performed using Ward’s minimum variance method. The results were presented using two complementary visualizations: a dendrogram and a heatmap. The dendrogram illustrates the hierarchical structure and the relative distances between the formed clusters. The heatmap provides a color-coded representation of the standardized (Z-score) performance of each genotype across all formations, with rows ordered according to the hierarchical clustering results to visually reinforce cluster separation. Cluster stability was assessed via permutation-based bootstrap resampling (pvclust, 1000 iterations), with clusters showing approximately unbiased (AU) *p*-values >95% considered statistically supported. Internal consistency of the clustering structure was further evaluated using silhouette analysis, with the average silhouette width serving as a measure of separation between genotype groups.

All figures—including bar plots, heatmaps, PCA biplots, dendrograms, scatterplots, and binary formation maps—were generated using R packages such as ggplot2 (version 3.5.1), factoextra (version 1.0.7), pheatmap (version 1.0.12), dplyr (version 1.2.5), and stats (version 4.5.1). Data was processed and visualized within the tidyverse framework, ensuring reproducibility and clarity. Graphs were formatted for publication, with standardized axis labels, legends, and color schemes to facilitate interpretation.

## 5. Conclusions

This study, for the first time, provides an integrated assessment of genotype-dependent regeneration in sunflowers, combining hierarchical clustering, correlation analysis, and temporal formation profiling. The results demonstrate that mid-stage formation—particularly Root 14D—is a reliable early predictor of organogenic success, offering a practical basis for genotype screening and protocol refinement.

Genotype-by-medium interactions revealed that S1 medium failed to induce root formation in any tested genotype, whereas R4 promoted earlier and more synchronized organogenesis—primarily via root development—in specific genotypes. The classification of genotypes into non-regenerators, moderate responders, and high regenerators provides a functional framework for targeted breeding and transformation strategies.

In the sucrose concentration trials, with the exception of minimal shoot initiation observed at 5% sucrose, no root or further shoot regeneration was detected in either experiment. Explants preconditioned on MS medium with varying sucrose concentrations (5%, 10%, 15%) failed to initiate organogenesis upon transfer to S1 or R4 media. Similarly, direct variation in sucrose concentration on S1 medium (5%, 10%, 20%) did not stimulate root or shoot development. These findings suggest that the tested sucrose levels, whether applied during preconditioning or directly within regeneration media, support callogenesis but are insufficient to trigger morphogenic differentiation. Additional hormonal or environmental cues may be required to overcome this developmental block. 

By integrating temporal, morphological, and statistical dimensions, this study establishes a scalable framework for overcoming regeneration bottlenecks in sunflower transformation, with direct applications in molecular breeding and CRISPR/Cas genome editing. Resolving the regeneration bottleneck will accelerate the development of transformation-competent lines, minimize resource loss, and enhance the efficiency of downstream genetic improvement pipelines. Beyond identifying predictive markers, this work lays the groundwork for the rational design of regeneration protocols tailored to genotype-specific responses. Although centered on sunflower, the analytical framework—combining temporal formation profiling with multivariate clustering—is readily adaptable to other recalcitrant species facing similar regeneration challenges.

## Data Availability

The original contributions presented in this study are included in the article. Further inquiries can be directed to the corresponding author.

## Appendix A

**Table A1:** List of Sunflower (*Helianthus annuus* L.) Lines Used in the In Vitro Regeneration Experiment.

Line	Primary Cluster Status	Role
NO-SU-12	High Regenerator	Candidate for transformation
AS-1-PR	High Regenerator	Candidate for transformation
HA-26-PR	Moderate Regenerator	Model genotype for tissue culture trials
HA-26-PR-SU	Moderate Regenerator	Inbred line/Comparative
HA-74	Moderate Regenerator	Inbred line
VLA-8-PR	Moderate Regenerator	Inbred line
DOP-32-08	Moderate Regenerator	Inbred line
FE-54	Moderate Regenerator	Inbred line
AB-OR-12	Moderate Regenerator	Inbred line
SOL-SU-26	Non-Regenerator	Inbred line/Recalcitrant
SC-MI-4-PR	Non-Regenerator	Inbred line/Recalcitrant
PR-2648-	Non-Regenerator	Inbred line/Recalcitrant
PH-BC2-91	Non-Regenerator	Inbred line/Recalcitrant
IMI-AB-14-PR	Non-Regenerator	Inbred line/Recalcitrant
HA-98	Non-Regenerator	Inbred line/Recalcitrant
AB-OR-8	Non-Regenerator	Inbred line/Recalcitrant
FE-7	Non-Regenerator	Inbred line/Recalcitrant Inbred line/Recalcitrant
OD-DI-65-SU	Non-Regenerator	Inbred line/Recalcitrant
